# Technological Evolution and Research Trends of Intelligent Question-Answering Systems in Healthcare

**DOI:** 10.3390/healthcare13182269

**Published:** 2025-09-11

**Authors:** Bingyin Lei, Panpan Yin

**Affiliations:** School of Management Science and Engineering, Beijing Information Science & Technology University, Beijing 100192, China

**Keywords:** AI, machine learning, large language models, natural language processing, medical chatbot, healthcare

## Abstract

**Background/Objective:** This study investigates the implementation and evolution of intelligent medical question-answering (QA) systems in healthcare to enhance service efficiency and quality. **Methods:** Through an integrated literature review and bibliometric analysis using CiteSpace 6.3.R1(64-bit) Basic software, we systematically evaluated core concepts, frameworks, and applications within medical QA systems, analyzing literature from 2018 to 2025 to identify research trends. **Results:** Significant applications were revealed across clinical decision support, medical knowledge retrieval, traditional Chinese medicine (TCM) formulation development, medical imaging report analysis, medical record quality control, mental health monitoring, and emotion recognition, demonstrating optimized resource allocation and service efficiency. Persistent challenges include system accuracy limitations, multimodal interaction capabilities, user trust barriers, and privacy protection concerns. **Conclusion:** Future research should prioritize multimodal diagnostic imaging, TCM-specific AI agents, and virtual-reality-assisted surgical exploration. **Contributions:** This work consolidates current achievements while establishing theoretical–practical foundations for innovation and large-scale implementation, advancing intelligent healthcare transformation.

## 1. Introduction

The rapid advancement of artificial intelligence (AI) and digital transformation in healthcare has intensified public demand for accessible medical services. Current expectations extend beyond traditional clinical models to encompass real-time health monitoring and in-depth medical knowledge acquisition. Nevertheless, China’s healthcare system remains under optimization, struggling to fully meet these demands. In this context, intelligent medical question-answering (QA) systems have emerged as a novel human–computer interaction paradigm with significant practical implications [1]. Given constraints in healthcare staffing and scheduling, developing such systems is critical. They effectively alleviate clinicians’ workloads by automating routine queries, enabling professionals to focus on complex medical tasks [2]. This innovation optimizes resource allocation, enhances service efficiency, and elevates care quality. By delivering rapid and accurate consultations, QA systems transcend spatiotemporal barriers, empowering users to access medical advice anytime, anywhere—thereby reducing healthcare burdens. Through continuous knowledge refinement, these systems provide personalized guidance that improves patient education and confidence, ultimately enhancing healthcare experiences.

To address China’s uneven distribution of medical resources, QA systems facilitate equitable access via online consultations, democratizing high-quality care [3]. Currently, AI serves as a pivotal force in governmental and industrial transformation. As a quintessential AI application, medical QA systems advance both technical capabilities and broader AI adoption. Consequently, they play an indispensable role in elevating healthcare quality, reducing costs, and accelerating AI progress.

However, existing research exhibits critical gaps: insufficient systemic analysis of QA frameworks and limited elaboration on healthcare-specific implementation details. To bridge these gaps, this study comprehensively examines the evolution of medical QA systems, analyzing key development aspects—evaluation metrics, data collection, knowledge storage, natural language understanding (NLU) modules, knowledge computation, and dialog management. We further systematize healthcare applications with case studies, aiming to establish theoretical foundations and practical guidelines for system refinement.

The innovation of this study is that it adopts a combination of literature review and bibliometric analysis and uses CiteSpace software to conduct in-depth mining and analysis of a large amount of related literature, so as to more comprehensively and accurately grasp the current research status and development trend of the intelligent medical QA system. In addition, this study will provide a systematic summary and outlook on the construction and application of intelligent medical QA systems from the practical needs of the healthcare field, so as to provide new perspectives and ideas for promoting the technological advancement and wide application of intelligent medical QA systems.

This paper is organized as follows: Firstly, the concept, definition, and framework of the QA system are introduced, then the evolution process of the QA system and its application in the medical field are elaborated in detail. Next, the construction of the QA system is discussed in depth, including relevant evaluation indices, data collection, knowledge storage, natural language understanding module, knowledge computing module, and dialog management and interaction. Then, a systematic summary of the intelligent QA system in the healthcare field is presented, analyzing it with typical cases, as well as a bibliometric analysis of existing studies. Finally, we conclude the whole paper and look forward to the future development trend of the intelligent medical QA system.

## 2. Question Answering Systems

### 2.1. Concepts, Definitions, and Frameworks

Question answering (QA) systems constitute a cornerstone of natural language processing (NLP), serving as advanced information retrieval frameworks engineered to deliver precise responses to natural language queries. Emerging during the 1950s within the framework of the Turing test, early systems, including BASEBALL (1961) [4] and ELIZA (1966) [5], established foundational techniques for domain-specific and conversational QA. These systems have evolved from text-limited paradigms toward comprehensive frameworks supporting multimodal interactions (e.g., images, audio, and video) [6,7]. For example, Microsoft Xiao-Ice [8] and Alibaba’s Xiaomei [9] demonstrate multimodal systems that integrate text, images, and video for e-commerce and social applications.

**Figure 1:**
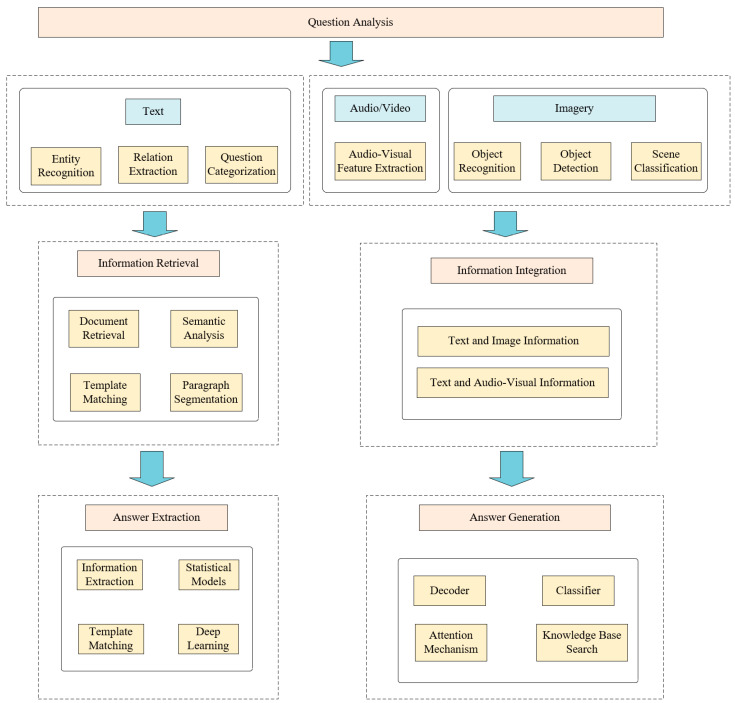
Processing framework for the QA system.

The QA structural framework comprises three core phases (Figure 1): question analysis, information retrieval, and answer extraction. Firstly, features are extracted from the multimodal information input by the user, such as text, image, audio, etc., and fused into a unified representation. Next, relevant data are retrieved and filtered from knowledge bases or document collections. Finally, concise answers are generated through template matching, semantic parsing, or deep learning techniques [10].

### 2.2. Evolution and Medical Applications

The evolution of QA systems is characterized by three distinct phases: The initial phase (1960s–1990s) was defined by rule-based systems, such as LUNAR (Geology) [11] and TREC QA Track [12]. Such systems relied exclusively on expert-defined rules and structured databases. The subsequent phase (2000s–2010s) featured data-driven systems, including START and Siri [13], which utilized open-domain architectures with free-text corpora and community platforms. The current phase (2020–present) is dominated by AI-driven paradigms employing large language models (e.g., ChatGPT [14]) and knowledge graphs to enable context-aware multimodal interactions.

In healthcare, medical QA systems are categorized as semantic-based systems or keyword-matching systems. Semantic-based systems integrate clinical guidelines and PubMed data for query parsing, with AskHERMES [15] being representative. Keyword-matching systems use shallow syntactic analysis but lack semantic depth, as exemplified by MedQA [16].

While systems such as MEANS [17] and AskCuebee [18] address specialized domains (e.g., parasitology), significant challenges persist in handling negation and complex reasoning. ChatGPT shows potential for medical triage but is limited by factual inaccuracies and instability [14]. HestiaQA [19] and Medicine Seekers emphasize disease-specific counseling importance yet exhibit challenges in maintaining answer consistency.

## 3. Construction of QA System

### 3.1. Relevant Evaluation Indicators and Data Collection

#### 3.1.1. Relevant Evaluation Indicators

Regarding the evaluation metrics of the QA system, the common metrics are accuracy, F1-score (F1), precision, recall, exact match (EM), mean average precision (MAP), and mean reciprocal rank (MRR).

Accuracy (Acc) is the ratio of the number of correct responses to the total number of questions, as in Equation (1):

(1) $$\mathrm{Accuracy}=\frac{TP+TN}{N}$$

Here, TP (true positive) denotes the number of samples correctly predicted as positive, TN (true negative) represents samples correctly predicted as negative, and N signifies the total test samples.

F1-score is a metric that combines precision and recall, as in Equation (2):

(2) $$F1=2\times \frac{\mathrm{Precision}\times \mathrm{Recall}}{\mathrm{Precision}+\mathrm{Recall}}$$

Precision is the percentage of correct answers among the answers provided by the QA system, as in Equation (3):

(3) $$\mathrm{Precision}=\frac{TP}{TP+FP}$$

FP (false positive) indicates samples misclassified as positive.

Recall is the ratio of the number of correct answers returned by the system to the number of all correct answers, as in Equation (4):

(4) $$\mathrm{Recall}=\frac{TP}{TP+FN}$$

FN (false negative) refers to samples misclassified as negative.

Exact match (EM) is a measure of the perfect match between the predicted answer and the actual facts, which is commonly used in the question and answer task data SQuAD, as in Equation (5):

(5) $$EM=\frac{Num_{right}}{Num_{total}}$$

The mean average precision (MAP) is the average of the mean precision of multiple questions, which evaluates the quality of the answer ranking returned by the system, as in Equation (6):

(6) $$MAP=\frac{1}{Q}\sum_{i=1}^{Q}A\mathrm{verage}\;\mathrm{Precision}(q_{i})$$

The mean reciprocal rank (MRR) is a measure of the quality of the ranking of the retrieval results, as in Equation (7):

(7) $$MRR=\frac{1}{\left|Q\right|}\sum_{i=1}^{\left|Q\right|}\frac{1}{rank_{i}}$$

In Equation (7), MRR is often used as a metric in multi-result problems, where Q denotes the total number of queries and $rank_{i}$ is the sequence of queries. Hits@K is the proportion of correct answers appearing in the first K returned results, as in Equation (8):

(8) $$\mathrm{Hits}@K=\frac{Num_{right}\;\mathrm{in}\;\mathrm{TOP}\;K}{Num_{total}}$$

In Equation (8), by adjusting the value of K, the performance under different numbers of returns in the QA system can be evaluated.

Bilingual evaluation understudy (BLEU) is suitable for evaluating the metrics of machine translation quality, which is widely used in the field of QA systems, and a higher value of BLEU indicates that the generated answer is more similar to the reference answer, i.e., the quality of the answer is higher, as in Equation (9):

(9) $$\mathrm{BLEU}=BP\cdot \exp\left(\sum_{n=1}^{N}\omega_{n}\log p_{n}\right)$$

where BP is the length penalty factor, $\omega_{n}$ is the weight for different N-grams, $p_{n}$ is the corrected precision, N-gram is a segment consisting of n consecutive words inside an utterance, and N is the segment length.

Recall-oriented understudy for gisting evaluation (ROUGE) is mainly based on the calculation of recall and is a measure of how much information from the reference answer is included in the generated answer.

#### 3.1.2. Data Acquisition

Publicly accessible natural language data are available in various forms [20,21]. These data can be transformed into large-scale, high-quality, diverse, and actionable datasets through de-duplication and cleaning processes, thereby establishing foundational databases for medical QA systems. Generic datasets possess rich training data and linguistic diversity, enhancing QA system performance and applicability. In addition, synthetic datasets generated from advanced language models (e.g., ChatGPT) have been utilized for model training. The synthetic data are widely adopted due to their low acquisition cost and high quality [22]. This innovative approach has yielded high-performance language models.

Medical QA systems typically leverage resources, including publicly accessible healthcare platform QA data, doctor–patient dialogs, knowledge bases, open medical datasets, and ChatGPT-generated synthetic data [23]. Table 1 summarizes prevalent biomedical datasets while analyzing their distinct contents and applications.

### 3.2. Knowledge Storage

The knowledge storage module stores extracted triples within a graph database while providing visual knowledge graph representations. Knowledge graph storage centers on triple-based systems, primarily encompassing RDF graph models and attribute-based graph databases, with the latter constituting the current mainstream approach. Related research addresses RDF graph data management [33,34], graph querying [35], and computational frameworks [36,37]. Reference [38] provides a systematic review of data models, query languages, and storage management, establishing theoretical foundations for knowledge storage development.

Three primary storage methods exist: relational databases, RDF-specific triple stores, and native graph databases. Relational databases employ mature strategies—including triple, horizontal, attribute table, and vertical partitioning storage—yet exhibit query performance limitations and significant storage demands. RDF-specific triple stores support SPARQL standards, offering formatting advantages. Native graph databases demonstrate superior storage/query efficiency, particularly for associative queries, with Neo4j representing the industry standard. Each method exhibits distinct tradeoffs, enabling flexible selection—particularly in medical knowledge graphs—of single or hybrid approaches based on application requirements, with critical attention to inter-format synchronization and conversion.

### 3.3. Natural Language Understanding Module

The natural language understanding module performs named entity recognition and intent recognition on user queries to extract semantic meaning. Early methods primarily utilized rule-based and statistical approaches. Rule-based systems depended on manually crafted grammar and expert knowledge, exhibiting limited scalability and adaptability to linguistic evolution. Subsequently, statistical methods, including N-gram models, emerged, processing linguistic data through word co-occurrence probability calculations. These methods demonstrated partial performance improvements but remained limited in capturing long-range dependencies and complex semantic relationships. In 2013, the Word2vec model was proposed by Mikolov, marking a milestone in natural language understanding. This model employs shallow neural networks to map words into low-dimensional vector space, where semantic relationships are represented through vector distances and orientations. Its two primary architectures—Skip-gram and CBOW (Continuous Bag of Words)—learn word vectors by predicting context words and center words, respectively. With advancing deep learning techniques, recurrent neural network (RNN) was introduced for natural language understanding. Long short-term memory (LSTM) [39] and gated recurrent unit (GRU) [40] mitigate RNN gradient vanishing through gating mechanisms, enabling stable and efficient long-sequence processing. Peters et al. [41] argued that context-aware pretrained vectors should encapsulate rich syntactic and semantic information, leading to the ELMo (Embeddings from Language Models) model development. In 2018, BERT (Bidirectional Encoder Representations from Transformers) was introduced by Devlin et al. [42], representing another breakthrough in natural language understanding. BERT adopts the transformer [43] architecture to capture contextual information through bidirectional training. Masked language modeling and next-sentence prediction tasks are employed during pretraining. Breakthrough results were achieved across multiple natural language understanding benchmarks. The pretraining–finetuning paradigm subsequently became standard for natural language understanding methodologies. Similarly, OpenAI’s GPT series [44] (Generative Pretrained Transformer) has demonstrated powerful language capabilities through large-scale pretraining. Recently, large language models, including ChatGPT [45], Meta’s LLaMA [46], Baidu’s Wenxin Yiyan, and Alibaba’s Tongyi Qianwen [47], have garnered significant attention for their contextual comprehension and text generation capabilities. Advancing natural language technologies are increasingly being deployed in high-risk, high-stakes, and sensitive domains. This trend presents new challenges and opportunities. Consequently, enhancing decision transparency while ensuring prediction credibility has emerged as a critical research focus.

Natural language understanding (NLU) technologies in healthcare have evolved along a generic NLP pathway yet demonstrate a distinct trajectory due to the specialized nature of medical texts. Early rule-based approaches relied on medical ontologies for structuring electronic health records (EHRs) but had poor scalability. N-gram models could mine drug associations but found it difficult to handle long-distance semantics. During the deep learning era, PubMed2vec established medical terminology embeddings, RNN/LSTM improved time-series medical data processing, and BioBERT and ClinicalBERT enhanced clinical decision support via domain-specific finetuning. Contemporary large language models represented by ChatGPT have been successfully adapted to medical data demonstrating efficacy in differential diagnosis and virtual patient consultation. However, stringent regulatory requirements in medical contexts present interpretability challenges, motivating efforts to resolve the “black box” dilemma through techniques like attention visualization.

### 3.4. Knowledge Computing Module

The knowledge computation module converts natural-language-processed interrogatives into structured queries, which are executed against the knowledge graph to retrieve answers. Knowledge graph technology serves as the core enabler for computable medical knowledge research, with key methodologies focusing on knowledge representation, named entity recognition and entity relationship extraction. Regarding knowledge representation, medical models have been constructed using rule bases, data-driven approaches, semantic triples, and ontologies, as evidenced by Gao et al.’s multilayer model for data compatibility optimization [48], Yang et al.’s EHR-based disease prediction framework enhancing structured knowledge computability [49], Suo’s proposition of semantic triples as fundamental representation units [50], and Patel’s demonstration of ontological efficacy in representing imprecise heterogeneous knowledge [51]. Knowledge representation methodologies evolve along two dimensions: feature-based approaches integrate textual content, attributes, and positional features (e.g., Ye et al.’s “knowledge pattern” framework [52] and Song’s adversarial learning integration of multicenter clinical data [53]); conversely, association-based methods utilize semantic triples and ontologies as foundational units [54]. Semantic ternary and ontology modeling depict inter-knowledge relationships, as demonstrated by Li et al.’s RDF-based clinical big data representation [54] and Zhang’s multimodal feature aggregation network for medical knowledge embedding [55].

Named entity recognition (NER) has evolved from dictionary-rule systems through traditional machine learning to deep learning, with contemporary dominance by LSTM-CRF and BiLSTM-CRF architectures [56,57]. For limited-data scenarios in Chinese clinical guidelines, low-resource NER research concentrates on data augmentation and model transfer techniques. In model structure design, lexicon-matching conflicts are resolved by Ding et al. via multigraph architectures [58], while lexical information integration through transformers enhances recognition efficiency (Li et al. [59]). Regarding data resource optimization, feature capture is enhanced by Sun et al. using AdaBoost [60], whereas lexicon weighting improves model inference (Liu et al. [61]). Entity relationship extraction identifies semantic links via co-occurrence analysis, rule-based systems, and machine/deep learning approaches. Deep learning methods exhibit superior performance: Chen et al.’s BiLSTM-CRF achieved 82.27% F1 on EHR relationships [62], PubMedBERT attained 92% F1 in low-resource biomedical extraction (Milosevic et al. [63]), and Cao et al.’s knowledge base fusion yielded 96.00% F1 for cancer mutation gene–disease relationships [64]. These approaches establish the foundation for structured healthcare knowledge processing and efficient implementation.

### 3.5. Dialog Management and Interaction

Early dialog management relied on rudimentary text interactions, wherein systems provided single responses post-user queries. This approach exhibited dynamic limitations and struggled with semantic associations due to inadequate contextual modeling for multi-turn reasoning. To enable natural conversational experiences </mark>, <mark> dialog strategies and interaction designs have been enhanced through multidimensional optimizations. For example, deep learning context modeling techniques (e.g., transformer and memory network) are employed to build a state tracking mechanism enabling implicit need identification via extended dialog history support. Reinforcement learning is integrated to optimize dialog policies, with systems trained through physician–patient scenario simulations to proactively disambiguate queries and dynamically adapt responses during multi-turn exchanges, thereby enhancing QA logic and user satisfaction.

Additionally, voice interaction technology is increasingly being implemented in medical QA systems. Speech recognition and synthesis technologies facilitate voice-based querying and response delivery, enhancing interaction naturalness and convenience. Concurrently, multimodal interaction has emerged as a research focus, enabling system integration of graphical inputs (such as uploaded laboratory reports and medication instructions) with voice data. Cross-modal fusion models aim to achieve composite “voice-to-image-to-text” interactions. Furthermore, medical scenario specificity is addressed through precise intent analysis: domain-specific classifiers distinguish diagnostic consultations, medication guidance, health science dissemination, and health information queries. Personalized responses are subsequently generated based on user profiles (e.g., age and medical history).

## 4. Intelligent Question Answering System in Healthcare

### 4.1. Intelligent Assisted Medical Decision Support

Medical-assisted decision-making represents a critical application scenario for intelligent medical QA systems. The diagnosis–treatment workflow is optimized through deep analysis of patient data, delivering critical information to clinicians. Accurate support is provided across diverse medical scenarios via intelligent discrimination and model integration within clinical decision support systems. At the data level, key diagnostic insights are generated through analysis of consultation records, electronic health records (EHRs), and medical image data. At the model level, optimal integration pathways between large language models and expert systems are identified to deliver scenario-specific decision support.

Intelligent medical QA systems demonstrate robust predictive and classification capabilities, enabling synthesis of personalized patient profiles with comprehensive medical datasets to generate individualized treatment plans. This customized clinical decision support enhances treatment outcomes for healthcare providers.

### 4.2. Medical Knowledge QA

Document data are segmented into discrete paragraphs via a paragraph-splitting model, establishing the foundation for subsequent text vectorization. These paragraphs are then vectorized using a text embedding model, with a document vector index library constructed to enable efficient retrieval. Upon query initiation, user inputs are vectorized by the QA system, followed by vector retrieval operations to identify maximally relevant document segments. Matching results are integrated with prompt templates, assembling inputs processed by the large language model to generate final responses. Final outputs are delivered via streaming API to user terminals, enabling rapid and accurate knowledge services. MedGPT, a healthcare-specific large language model, delivers detailed medical condition explanations, treatment plan recommendations, and common health problem resolutions through conversational interactions. Leveraging multi-turn dialog training datasets, it provides accessible health intelligence services.

### 4.3. Chinese Medicine R&D and Pharmacy Services

Intelligent QA system implementation in pharmaceutical research enhances efficiency, reduces costs, improves accuracy, and enables personalized treatment planning, representing a significant industry trend. In traditional Chinese medicine (TCM) and pharmaceutical services, QA system modules integrate modern pharmacology with TCM principles to analyze herbal constituents in depth, identifying potential herb–drug interactions and safety concerns. Building on Chinese medicine canon and drug interaction research, novel personalized treatment concepts and tools are generated. Computer simulations enable prediction of drug activity profiles, safety parameters, and adverse effects.

QA systems have facilitated breakthrough innovations in cardiovascular and antineoplastic therapeutics. In 2015, two Ebola-inhibitory drug candidates were identified by Atomwise using AI algorithms, reducing discovery timelines by orders of magnitude. Traditional methods may take months or even years to achieve similar results. Therefore, AI-based drug screening demonstrates significantly higher efficiency versus conventional techniques [65]. Multimodal QA systems analyze chemical and biological data during development, predicting compound bioactivity, toxicity, and pharmacokinetic properties. This capability enables discovery of novel drug targets, disease mechanisms, and therapeutic strategies. TCM pharmaceutical services extend QA system capabilities through drug knowledge base integration, enhancing medical query resolution. After receiving questions submitted by users, the service first calls the drug name entity recognition module to extract drug names, then calls the text vectorization model to vectorize the extracted drug names and retrieves the standard drug names from the drug name vector library to obtain the drug primary key. User queries are then converted to semantic vectors, triggering retrieval from drug specification vector libraries and related literature. External knowledge is integrated with queries to assemble prompts, which are processed by the QA system for answer generation.

Retrieval-augmented generation technology integrates semantic search and generative mechanisms, enabling rapid extraction of precise knowledge from pharmaceutical databases and synthesis of clinically accurate responses. These tightly integrated retrieval and generation systems exhibit enhanced accuracy and response speed, revolutionizing pharmaceutical knowledge acquisition and user experiences.

### 4.4. Medical Imaging Report Analysis for Accurate Diagnosis and Treatment Decision-Making

Diagnostic imaging in medicine can assist doctors in accurately diagnosing and treating patients’ conditions; however, traditional methods are prone to issues, such as highly repetitive work, which is prone to increasing the diagnostic error rate, prolonging diagnostic time, the absence of an auditing doctor for certain medical imaging categories, and a stronger reliance on individual doctors’ experience. The multimodal intelligent QA system can develop an image report analysis service that enables doctors to perform preliminary image screening, conduct quantitative disease analysis, and generate disease analysis reports by incorporating big data analysis and AI deep learning technology. The service automatically analyzes medical images through AI reading, identifies the patient’s lesion area, and generates a detailed image report that includes the lesion’s location, size, and morphological features, as well as an accurate diagnosis based on the analysis results. For follow-up patients with a history of multiple imaging examinations, the system can automatically retrieve the patient’s historical report information for the same anatomical region, conduct detailed comparative analysis of the reports, correlate the relevant diagnostic information, and generate diagnostic imaging conclusions.

The image report analysis service has three specific application scenarios: guided diagnostic conclusions, comparative analysis scenarios, and fully automated generation services. Guided diagnostic conclusions enable the image report analysis service to automatically generate diagnostic conclusions based on the imaging doctor’s textual description of the findings. Doctors can then use the automatically generated content to adjust their references and produce a complete image report. Comparative analysis scenarios allow the system to retrieve historical reports of patients with multiple imaging tests, enabling a comparative analysis of the findings across different reports and generating a diagnosis that reflects both the current imaging results and historical trends. A fully automated generation service streamlines the entire diagnostic process, from AI reading and image description to the final diagnostic conclusion.

### 4.5. Quality Control Analysis of Medical Record Content

Quality control (QC) of medical records, the core content of hospital quality management, can detect and correct errors and defects in the medical process, ensuring the accuracy, completeness, and reliability of medical information [66].

Currently, most medical institutions have established electronic medical record quality control systems to ensure timeliness and completeness. However, these systems primarily monitor the formal aspects of quality control, with much of the content still relying on manual sampling. Quality control officers need to review numerous medical records, orders, and examination reports, a process that is labor-intensive and inefficient. Moreover, manual sampling can only cover a limited portion of a hospital’s medical records. The intelligent QA system’s medical record content QC analysis applies to multiple medical scenarios, shifting traditional rule- and template-based QC methods toward self-learning models. This enables the system to grasp the complex nuances of QC rules and ensures logical medical record content checking. By finetuning the model, the large language model can better understand human language requests and provide detailed and accurate explanations of the quality control results, instead of merely returning “yes” or “no” to indicate compliance with the quality control rules. According to the type of QC rules, different algorithmic model levels are automatically matched to address automated content QC, self-explanatory content QC, and self-learning feedback mechanisms, enabling the model to better satisfy diverse user needs during self-learning.

### 4.6. Mental Health Monitoring and Emotion Recognition

Recent advances in AI-driven mental health monitoring have shifted depression detection from subjective assessments toward objective quantification. Facial expression analysis—a core methodology—integrates multimodal data through deep learning models, emerging as a pivotal tool for identifying depression and affective disorders.

Convolutional neural networks (CNNs) now enable precise capture of depression-related micro-expressions. Pretrained models (e.g., VGG-19 and ResNet-152) achieve superior performance via transfer learning on datasets like CK+ and FER2013. Notably, VGG-19 attained 0.98 mean accuracy on CK+, establishing a technical foundation for early depression screening [67]. These models selectively freeze low-level feature extraction layers while finetuning high-level classifiers to detect subtle facial muscle dynamics (e.g., drooping corners of the mouth and periorbital muscle tension). Augmentation techniques (image rotation and brightness adjustment) further enhance robustness against real-world variables like lighting variations and facial occlusions [67].

Multimodal data fusion expands clinical applications. Integrating facial dynamics (expression duration and intensity variation), body posture (shoulder/neck rigidity), and vocal prosody elevates detection accuracy by 15–20% [68]. For instance, DenseNet architectures combining facial landmark trajectories with head-motion features comprehensively capture affective blunting in depression patients, including delayed emotional responses and reduced movement amplitude [68]. Such frameworks are now embedded in telemedicine platforms (e.g., DoctorLINK), enabling real-time emotion monitoring for remote psychological interventions [67].

Currently, the research in this field is developing toward the direction of ‘precision individualization’. On the one hand, the cross-group recognition bias is reduced by transfer learning to adapt facial expression features in different cultural contexts (e.g., more introverted emotional expression in Eastern populations); on the other hand, multimodal fusion models combining physiological signals (e.g., heart rate variability and skin electrical activity) and facial expressions are becoming a new path to resolve the neurophysiological mechanisms of depression [69]. These advances not only promote the automation and objectivity of depression detection but also provide a technological paradigm for achieving the full-cycle mental health management of ‘screening–intervention–follow-up’ by embedding the tele-diagnosis process [67].

## 5. QA System in Healthcare Field

### 5.1. Shenglang AI Clinical Intelligent QA System

Leveraging deep learning and natural language processing (NLP) technologies, the Shenglang AI Clinical Intelligent QA System constructs a multisource knowledge base integrating medical literature, clinical guidelines, and real-world cases. Text-based interactions and visual outputs are supported, including symptom analysis reports and treatment pathway diagrams. Diagnostic logic is optimized through patented algorithms, reducing misdiagnosis risk by 37%, as validated using clinical data from over 500 partner institutions, including the 301 hospitals. Dual-user interfaces serve clinicians and patients: patient-facing features enable symptom self-assessment, medication guidance, and triage advice, while clinician tools support diagnostic and therapeutic decision-making with real-time evidence-based recommendations and personalized treatment plans. The technical architecture achieves dynamic clinical data–knowledge graph fusion, demonstrating rare diseases’ characteristic identification in complex case analyses. This capability will be integrated into medical school curricula from 2025, exemplifying AI-enhanced clinical decision-making.

### 5.2. Zhiyun Health’s Knowledge Graph-Based Medical Intelligent Consultation System

A large-scale knowledge graph encompassing 3000+ diseases, 20,000+ drugs, and 1 billion electronic medical records has been developed by Zhiyun Health. Accurate consultation and prescription compliance verification are enabled through a rule engine and machine learning algorithms, achieving 99.97% accuracy in detecting drug contraindications. The system is differentiated in retail pharmacy and medical institution scenarios: the SaaS platform for 228,000 pharmacies provides real-time early warning of medication risks and improves the safety of medication at the grass-roots level, while the pre-inquiry module at the hospital end improves the efficiency of the outpatient process by 40% and innovatively supports the intelligent analysis of unstructured data, such as TCM symptoms and constitution identification, etc. Integration with the DeepSeek-R1 enhanced model is scheduled for 2025, with deployment in electronic medical records. Following implementation, abnormal indicator identification speed will increase 5-fold in electronic medical records, while chronic condition oversight in primary medical institutions will decrease to 1.2%. These enhancements will establish the system as a critical intelligent support tool within hierarchical diagnosis frameworks.

### 5.3. MedAI

MedAI focuses on intelligent solutions for the entire pharmaceutical R&D lifecycle. Its core functionalities encompass clinical trial management and medical data standardization. Utilizing natural language understanding (NLU) technology, its medical coding system automatically maps unstructured data to international standard terminologies, such as MedDRA and WHO-DD, with 98.7% accuracy, significantly enhancing clinical trial data processing efficiency. In new drug R&D scenarios, companies such as Hengrui Pharma and WuXi AppTec are assisted in shortening R&D cycles by 15–20% through protocol design optimization and automated CRF generation. Scheduled for launch in 2024, Version 3.0 integrates a real-world data (RWD) analysis module. This module mines drug efficacy signals from electronic medical records and healthcare insurance data, providing data-driven support for post-marketing safety monitoring and indication expansion, thereby establishing it as a key component for pharmaceutical industry innovation.

### 5.4. Pearl River Hospital DeepSeek Intelligent QA Platform

The Pearl River Hospital DeepSeek platform, based on DeepSeek large model architecture, supports localized deployment and multimodal interaction (voice input and image data parsing). It focuses on enhancing electronic medical record intelligence and improving primary care quality. Technically, its medical record generation module reduces structured documentation time from 2 h to 10 min, achieves 85% accuracy in image report logic verification, and enables automatic TI-RADS grading determination for thyroid nodule ultrasound reports (91% compliance rate). Following deployment in institutions such as the Gansu Linxia Maternal and Child Health Hospital, the platform’s medical record quality control system increased high-risk pregnancy guidance accuracy to 92%, reduced test report interpretation time by 93%, and lowered omission rates from 15% to 6%. Through the domain adaptation optimization of the large model, the platform has demonstrated its comprehensive processing capability for multimodal data in complex clinical scenarios and has become an important technical carrier for promoting the syncing of high-quality medical resources.

### 5.5. WiNGPT

WiNGPT, a medical-domain-specific large model (7 billion parameters), provides an intelligent service system covering the entire pre-diagnosis, diagnosis, and post-diagnosis process. Core technologies include multi-round consultation logic modeling (98% critical value recognition accuracy), structured medical record generation (efficiency improved by 3–5 times, error rate reduced to 2%), and personalized follow-up plan formulation (diabetic patients’ adherence improved by 27%). A key advantage is its support for integrated Chinese–Western medicine diagnosis. The traditional Chinese medicine (TCM) evidence identification model digitally reconstructs the TCM consultation process (95% accuracy). Additionally, the open-sourced 7B lightweight version (>100,000 downloads) promotes AI-assisted diagnostic tool adoption in primary hospitals. In the pilot studies of the People’s Hospital of Peking University and Zhejiang Provincial Hospital of Traditional Chinese Medicine, the system has shortened the average outpatient consultation time by 22 min, and the standardization rate of medical record writing has increased from 68% to 94%, which has become a benchmark system of medical informatization construction that is both technologically innovative and practical on the ground.

### 5.6. Comparative Analysis of Healthcare Question-Answering Systems

To systematically profile medical QA systems, we conducted a comparative analysis of five mainstream platforms. As summarized in Table 2, Shenglang AI and Zhiyun Health emerged as the most widely adopted solutions due to their comprehensive coverage of clinical decision-making and primary care demands.

## 6. Challenges and Future Trends

### 6.1. Problems and Challenges

#### 6.1.1. Accuracy and Professionalism

Although the intelligent medical QA system possesses a certain degree of general medical knowledge, it has significant limitations both in terms of specialized medical knowledge reserves and serious medical numerical or intellectual reasoning. As a result, the system may generate inaccurate or even incorrect medical advice during the dialog process [70], which severely limits its application in medical tasks with high difficulty, depth, and real-time requirements.

The essence of large language models (LLMs) lies in generating evidence-based judgments and predictions. Thus, maximizing their potential in healthcare hinges critically on overcoming data bottlenecks. Compared to vertical-domain LLMs, medical data’s specialized, complex, and heterogeneous nature creates unique deployment challenges: low data quality, severe data siloing, impeded data assetization, and stringent demands for multimodal data synergy, standardization, and timeliness.

First, medical data span diverse diseases, patient cohorts, and clinical scenarios. Multimodal sources—text, images, and genomic data—originate from disparate healthcare institutions and information systems, resulting in inconsistent data quality and high integration complexity. Second, the absence of unified data standards and interoperable interfaces compounds these issues. Most hospitals restrict database access to local networks, perpetuating data silos that obstruct LLM training.

Model hallucination constitutes a major safety barrier for medical LLMs. This phenomenon occurs when models generate content based on internal constructs rather than factual data, manifesting as fabricated details or erroneous factual interpretations. As Zheng Haihua (CTO of WeMed) notes, the non-transparent algorithmic processes underlying LLM reasoning raise concerns about explainability and reliability in clinical applications. The Taxonomy of LLM Hallucinations (Tencent AI Lab) classifies hallucinations into three categories:

1. Input-conflicting hallucinations: incongruent responses (e.g., describing disease symptoms when queried about treatments).
2. Context-conflicting hallucinations: inconsistent outputs across sessions (e.g., diagnosing a cold today versus allergies tomorrow for identical symptoms).
3. Fact-conflicting hallucinations: fabrication of pseudo-factual content (e.g., misidentifying metoclopramide as “natural gas ketone” [71]).

Such hallucinations risk propagating misinformation, misdiagnosis, and clinical errors, endangering patient safety. Gartner’s July 2024 report cautions that generative AI in healthcare has entered the “trough of disillusionment”. Ensuring medical LLMs remain both effective and rational—preventing uncontrolled deviations—requires sustained efforts [71].

#### 6.1.2. Multimodal Interactivity

Current intelligent medical QA systems mainly rely on textual dialogs, while users may use multimodal medical data involving, e.g., text, speech, images, videos, and histological data, such as electrocardiograms, imaging reports, wound pictures, and exercise videos, in actual medical scenario dialogs [72]. These data are pivotal for disease diagnosis, precision medicine, and drug development. Yet realizing their full potential necessitates complex multi-stage data governance—a process critically challenged by the heterogeneity and complexity of multimodal healthcare data [73]. Healthcare data governance encompasses data acquisition, standardization, and integration. Data acquisition aggregates voluminous and diverse medical sources, standardization cleanses, transforms, and normalizes heterogeneous multimodal data into consistent formats for downstream processing, and integration consolidates these standardized datasets to create unified views enabling cross-source interoperability.

Existing governance frameworks exhibit limitations in multimodal data handling. Structurally, divergent semantic features across modalities complicate standardization and integration. Analytically, current tools predominantly process unimodal data, failing to leverage multimodal information holistically. Ethically, safeguarding confidentiality, integrity, and availability of sensitive health data while preventing breaches remains a persistent challenge [74].

Medical imaging analysis—spanning lesion detection, segmentation, and classification—faces analogous hurdles. Traditional manual methods are labor-intensive and subjective, increasing misdiagnosis risks. AI models trained on specialized datasets require costly expert annotations and complex feature extraction yet demonstrate poor generalization across institutions and imaging devices [75]. Addressing these issues mandates resolving multimodal data fusion barriers to securely integrate imaging, pathology, genomics, and EHRs, thereby advancing data assetization. Developing medical semantic data cleaning/annotation frameworks will provide high-quality structured training data, facilitating EHR sharing, test result interoperability, and optimized insurance services.

The smart hospital era transforms clinical workflows and operational paradigms. Embedding large models throughout diagnostic–therapeutic processes necessitates enhancing clinicians’ AI interaction skills and investigating foundational theories: personalized learning, multi-agent collaboration, and uncertainty reasoning. Establishing medical LLM training platforms [76], redesigning human–AI collaboration, and implementing adaptive clinician–patient–AI systems are essential to evolve from diagnostic assistance to closed-loop therapeutic decision-making.

#### 6.1.3. User Adherence and Trust Issues

Despite preliminary applications of medical large language models (LLMs) in drug discovery, patient consultation, medical imaging decision support, and health management, fragmented scenario-specific requirements and insufficient domain comprehension perpetuate trust deficits—a primary barrier to widespread adoption [71]. As healthcare constitutes a credence good, users cannot evaluate service quality through experience or cost alone, relying predominantly on trust-based selection. Consequently, patients preferentially rely on clinicians’ expertise over algorithm-generated diagnoses despite continuous LLM performance improvements, explaining limited real-world acceptance [71].

Furthermore, while LLM accuracy advances (e.g., Tencent Health AI’s 87% accuracy in medical note summarization), significant performance gaps persist in core diagnostic tasks compared to experienced physicians. Studies confirm limitations in treatment planning, where even minor errors risk severe consequences—fostering clinician and patient caution toward LLM-driven decisions [71]. In case of intellectual errors or misleading information, patients’ compliance may significantly decrease, and physicians may question their reliability [77]. In addition, there are significant differences in the acceptance of chatbots among users of different regions and age groups: users in developed regions are more likely to accept them due to higher technology penetration, while users in developing regions are relatively conservative, and young users tend to accept technological innovations, while older users have higher thresholds due to technological unfamiliarity and operational complexity [78]. Therefore, improving the credibility and transparency of smart medical QA systems and optimizing their design for different user groups are key challenges to increase their acceptance and long-term application value.

#### 6.1.4. Privacy Protection and Ethical Issues

Intelligent medical QA systems face serious privacy and security risks when processing user data. Despite the gradual maturation of data encryption and anonymization technologies, the possibility of privacy leakage still exists [70]. In addition, errors in medical advice may lead to serious consequences, and the issue of responsibility attribution needs to be clearly defined [79]. Despite data’s established status as the fifth production factor, medical data assetization confronts persistent barriers. Hospitals adopt conservative approaches toward cross-institutional data sharing due to stringent privacy requirements, high misuse risks, ambiguous accountability frameworks, insufficient trusted health data exchange platforms, and inadequate regulatory mechanisms for data circulation. Given that high-quality data constitute the foundation of LLM optimization, these constraints severely impede medical LLM training [71]. Therefore, it is important to develop strict regulatory policies and ethical standards to secure user data and maintain system reliability.

### 6.2. Future Development Trends

This study’s projections of future trends derive from bibliometric analysis, recent advancements in high-frequency AI technologies, and synthesis of domain-expert literature, encompassing multimodal diagnostic imaging, TCM-oriented intelligent agents, and virtual agents.

#### 6.2.1. Multimodal Image Diagnosis

Exploring diagnostic imaging based on multimodal AI techniques refers to the use of multiple types of medical data (e.g., images, text, sound, etc.) for training and modeling in order to provide accurate diagnosis, prediction, and treatment recommendations for a specific disease or medical scenario [80]. Numerous multimodal datasets integrate imaging studies with diverse data sources (e.g., genomics, clinical records, laboratory tests, and wearable devices). Through self-supervised learning, generic models applicable to multiple data types are generated, revealing relationships between image features, specific genomic signatures, and laboratory findings. Consequently, multimodal AI technology enables exploration of diagnostic imaging applications focused on early cancer biology, including cancer risk assessment and biomarker discovery/validation. By integrating various types of data, such as X-rays, pathology reports, and patient histories, and by using deep learning and machine learning algorithms to identify the type, grade, and prognosis of cancers, the combination of different types of data will provide a more comprehensive understanding of the patient’s condition and health status to provide more reliable support for medical decision-making [81,82,83].

#### 6.2.2. Intelligent Body of Chinese Medicine

Intelligent agents establish a framework enabling healthcare models to make dynamic decisions, thereby enhancing their capacity to address complex tasks and diverse scenarios. This foundation facilitates the transition of healthcare models from language-based systems to real-world applications.

In healthcare, large model-based intelligent agents will serve as collaborative partners across all treatment domains. For doctors, intelligent systems will provide a full range of services in their daily work. For instance, during outpatient encounters, these agents can record conversations in real time, automatically generate clinical documentation, provide updated clinical guidelines, and deliver research evidence to support treatment planning. In addition, they facilitate interdisciplinary expert coordination, optimize complex case management, and enable remote patient communication. During symptom analysis and preliminary diagnosis, patients are guided through appointment scheduling and pre-visit preparation [84,85].

The traditional Chinese medicine (TCM) intelligence agent integrates specialized domain knowledge to ensure delivery of high-quality clinical advice and diagnostic solutions. Enhanced by expert knowledge, this agent applies complex TCM theories with greater accuracy for patient treatment. Through analysis of extensive TCM literature and clinical data, evidence-based diagnostic and therapeutic plans are generated. For example, analysis of Chinese medicine formula efficacy enables optimization of treatment plans based on real-time patient responses. During treatment, medical recommendations are documented while patients receive plan clarification; during rehabilitation, medication management and health guidance are provided, including long-term monitoring for chronic conditions [86]. This all-round support reduces the burden of patients, on the one hand, and ensures the efficiency of the medical process on the other hand, creating a more connected, efficient, and humanized healthcare environment for doctors and patients together.

#### 6.2.3. Virtual Reality Surgery

Virtual reality surgical simulation constructs an operating room environment using patient-specific 3D anatomical models and frame-rendering technology. This enables a VR–environment interaction where instruments can be manipulated as in actual surgery, while patient images, records, and diagnostic data are accessible intraoperatively. However, current platforms exhibit significant limitations requiring enhancement. For example, it has not yet realized a more realistic simulation of the impact of surgical operations on patients’ physiological parameters, as well as failing to simulate the impact of differences in different patients’ conditions and other changes in the physical environment.

AI and large language model advancements will enhance virtual surgery interactivity and decision support. Using large language models and expert knowledge augmentation techniques, these deficiencies can be remedied through more refined data modeling and algorithm design. Specifically, surgical data analysis through machine learning enables generation of complex biomechanical models simulating patient-specific intraoperative physiological responses. Additionally, expert system integration provides real-time feedback for surgical optimization during procedures [87]. Large model-driven NLP technology facilitates real-time medical knowledge base queries, expert advice retrieval, and transformation of complex terminology into actionable surgical guidance. In addition, AI can help generate personalized patient data to better simulate the differences in different patients’ conditions. Real-time analytics enable dynamic intraoperative parameter adjustment. For instance, digital twin technology monitors physiological changes, providing feedback that—when integrated with large model knowledge bases—enhances surgical risk prediction and strategy adaptation. This combination not only improves the realism of virtual surgeries but also enhances their guiding role in actual surgeries. Leveraging large language model knowledge enhancement, global expertise is integrated into virtual platforms. During surgery, doctors can access best practices and expert advice for the current surgical situation in real time. This dynamic knowledge service enhances virtual surgery utility and bridges skill gaps between experienced and novice surgeons.

## 7. Bibliometric Analysis

The study of intelligent QA systems in healthcare has become a much talked about research topic. Relevant literature on intelligent medical QA systems published in SCI-indexed journals between 1 January 2018 and 31 March 2025 was reviewed and analyzed using CiteSpace software [88]. The literature search employed “healthcare” and “QA system” keywords to ensure comprehensive coverage of field findings.

### 7.1. Methodology and Data

#### 7.1.1. Methodology

CiteSpace software (Java platform), a powerful bibliometric visualization and document analysis tool, was utilized for in-depth analysis [88]. With robust bibliometric analysis capabilities, CiteSpace visualizes complex literature associations through scientific knowledge graphs, facilitating both historical research evolution tracing and future research direction prediction [88]. Its core capability involves transforming large-scale scientific literature into intuitive visualizations, incorporating efficient analysis modules for pathfinding, clustering, and timeline analysis [89]. Specifically, the pathfinding function identifies the shortest connection paths between nodes, revealing potential research topic associations and clustering groups nodes by similarity to identify academic subfields or research domains, while timeline analysis tracks field development, highlighting seminal milestones and key events [89]. In addition, CiteSpace also provides quantitative indicators, such as the frequency of citations and the intensity of author collaboration, which provide a scientific basis for researchers to assess the importance and influence of each node in the literature network [90].

#### 7.1.2. Data

This study used Web of Science (WOS) to collect literature data on the application of QA systems in healthcare over a period of 8 years (2018–2025). There were 509 records in the WOS core database, excluding review articles. This study selected “healthcare” and “Q&A system” as retrieval keywords based on core research objectives in medical QA systems, combined with high-frequency terms and domain hotspots from existing literature. Preliminary screening of publications validated keyword coverage, ensuring comprehensive representation of applications, technological evolution, and research trends for intelligent QA systems in healthcare.

### 7.2. Analysis of Results

**Figure 2:**
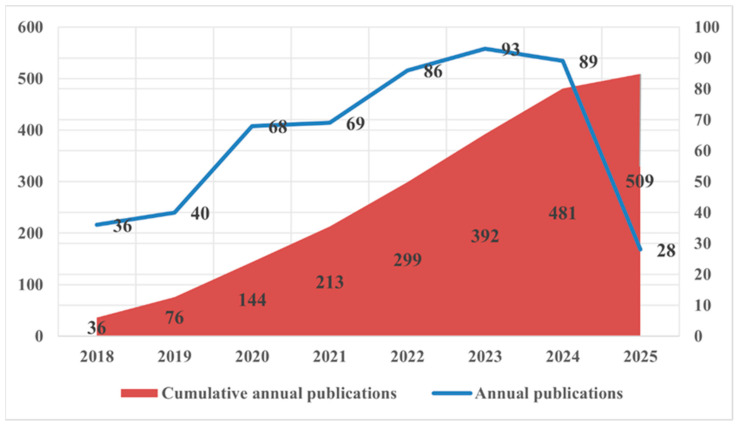
Annual distribution of the number of publications.

This study used the CiteSpace tool to synthesize and analyze relevant information from different countries, institutions, journals, and scholars and compare their contributions in the field of medical QA systems. As illustrated in Figure 2, 509 publications related to healthcare QA system applications were identified. From 2018 to 2024, there was a significant increase in the number of related publications, from 36 to 481. The cumulative annual publication count demonstrated a pronounced upward trajectory, indicating growing scholarly attention to healthcare QA system research.

#### 7.2.1. Countries and Publishers

**Figure 3:**
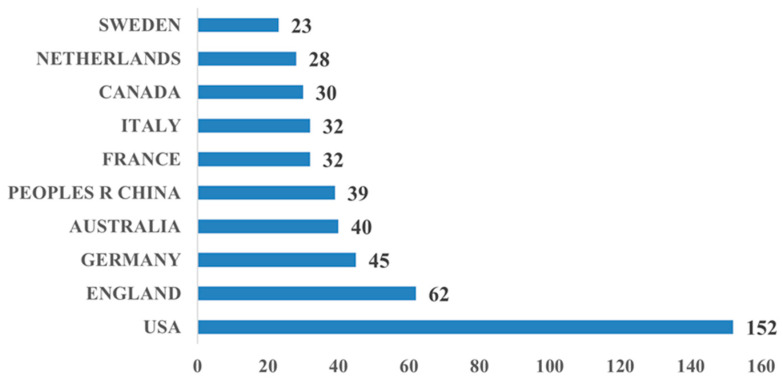
Bar chart of top 10 countries by number of publications.

As shown in Figure 3, the countries that have published the most papers on the application of QA systems in healthcare are the United States (152 papers), the United Kingdom (62 papers), Germany (45 papers), Australia (40 papers), and China (39 papers). The United States has the highest number of publications in this area, which is 2.45 times higher than the second-ranked country, the United Kingdom, indicating that the United States attaches the highest importance to the application of QA systems in healthcare. Consistent with the Pareto principle (80/20 rule), the top 10 countries contributed 94.9% (483/509) of publications, indicating that analyzing this dominant cohort efficiently reflects the field’s primary research contributors. We, therefore, focused on these 10 nations for subsequent analysis.

**Figure 4:**
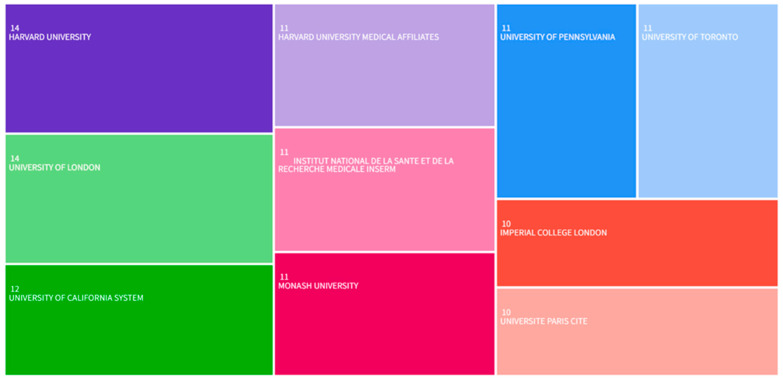
Tree diagram of the top 10 organizations by number of publications.

As presented in Figure 4, the most prolific institutions publishing on healthcare QA system applications were: Harvard University (14 article), University of London (14 articles), University of California (12 articles), Harvard University Medical Affiliates (11 articles), Institut National de la Sante et de la Recherche Medicale Inserm (11 articles), Monash University (11 articles), University of Pennsylvania (11 articles), University of Toronto (11 articles), Imperial College London (10 articles), and Universite Paris Cite (10 articles). Four of the top seven institutions are located in the United States. This distribution indicates substantial US research engagement in this domain. In addition, it also shows that the US has done a lot of work and extensive efforts in developing medical QA systems compared to other countries.

#### 7.2.2. Authors

**Figure 5:**
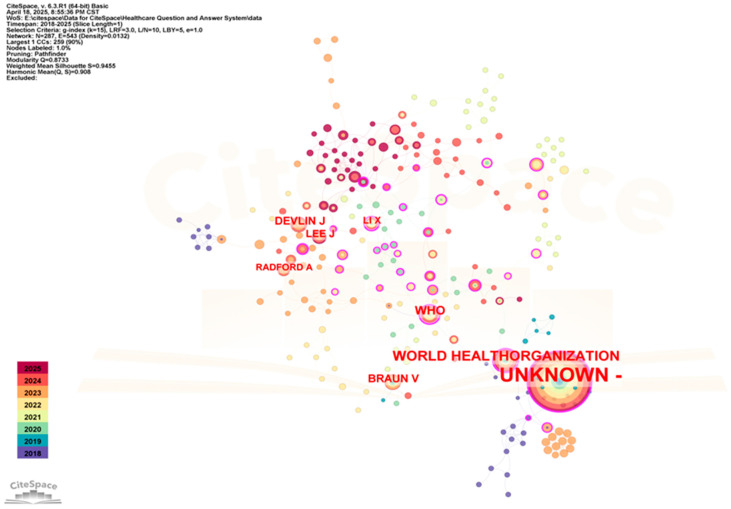
Co-citation network map of authors in related fields (time span: 2018–2025; slice length = 1; G-index = 15; LRF = 3; LBY = 5; e = 1; N = 287; E = 543).

In order to further analyze the research related to the application of QA systems in healthcare, this paper used CiteSpace to statistically summarize the authors of published studies and identify several influential authors in the field, as shown in Figure 5.

As depicted in Figure 5, the network comprised 287 nodes and 543 links with a density of 0.0132. Devlin J was cited 18 times, followed by Lee J with 17 citations.

These results indicate that Devlin Jacob is the most influential scholar in medical QA systems, with research focused on deep learning techniques for natural language processing enhancement. The BERT model (Bidirectional Encoder Representations from Transformers), proposed by Devlin et al. [42], has significantly advanced QA system research and development. The second ranked author is Joon Lee, who focuses on data mining of clinical data. The research of these authors represents the prevailing trend in the application of QA systems in healthcare.

#### 7.2.3. Topical Trends

**Figure 6:**
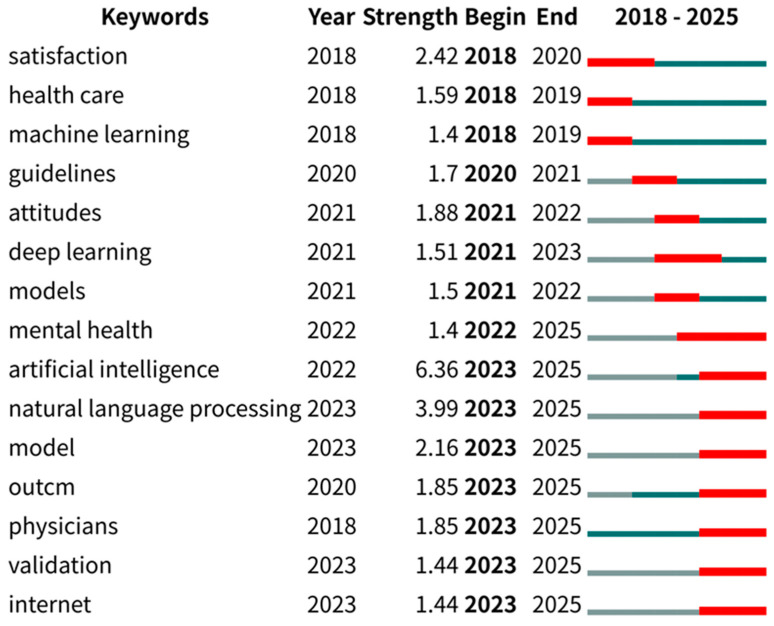
Annual thematic trend analysis.

Keyword emergence is defined as a rapid increase in keyword frequency over a short period, reflecting research hotspots that attract significant attention within specific fields and timeframes. In this study, CiteSpace was used to statistically measure topic trends over an eight-year period. Topic trends were assessed through keyword emergence, measured by frequency changes. Emergence intensity, calculated via a statistical formula, quantified this phenomenon, as illustrated in Figure 6.

As illustrated in Figure 6, temporal trends in healthcare QA system applications over the past eight years are depicted by the right-hand lines. During 2018–2020, “satisfaction”, “healthcare”, “machine learning”, and “guidelines” emerged as prominent keywords. This reflects the early trend hotspots for research on the application of QA systems in healthcare, with studies during this period discussing the use of big data technologies to improve decision-making and practice in the current state of healthcare. Between 2020 and 2022, “attitude”, “deep learning”, “modeling”, and “mental health” were identified as dominant keywords. This suggests that during this period, researchers were increasingly focusing on new computer technologies and models, and were paying more attention to mental health as a specific healthcare area. During 2022–2025, “artificial intelligence”, “natural language processing”, “models”, “outcomes”, “physician”, “validation”, and “internet” gained prominence, suggesting that when applying QA systems in healthcare, the researchers were placing more emphasis on emerging technologies and models in the field of AI, and that validation of healthcare technologies, data, models, and interventions was increasingly emphasized.

#### 7.2.4. Cited Journals

**Figure 7:**
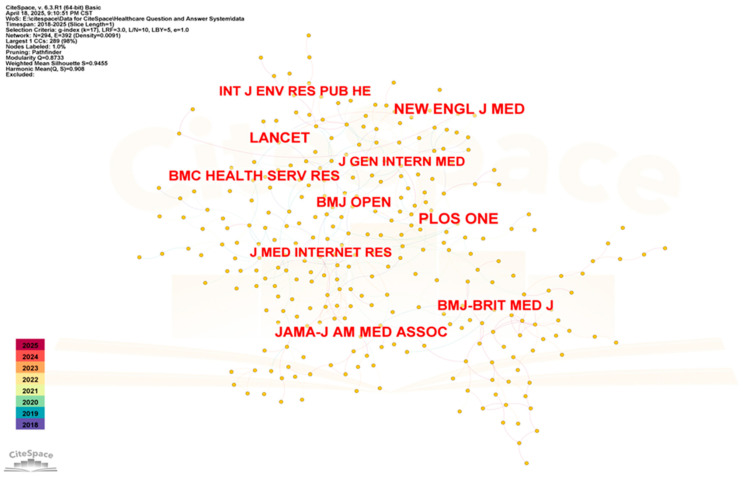
Co-citation network map of cited journals (time span: 2018–2025; slice length = 1; G-index = 17; LRF = 3; Lby = 5; E = 1; N = 294; E = 392).

To identify authoritative journals and understand research dynamics, cited journals in healthcare QA system research were analyzed using CiteSpace. As presented in Figure 7, there were 294 nodes and 392 connections.

*PLOS One* (125 citations) was the most cited journal—an open-access publication spanning life sciences, medicine, engineering, and computer science. *The Lancet* (111 citations), an internationally recognized premier integrative medicine journal, is dedicated to advancing scientific knowledge for societal benefit. *The New England Journal of Medicine* (110 citations) is a weekly general medicine journal, which publishes new research, review articles, and editorials on a range of topics of importance to healthcare, including a series of journals focused exclusively on medical informatics and the application of artificial intelligence to medicine. These highly cited journals constitute essential references and have profoundly influenced intelligent healthcare QA system research.

#### 7.2.5. Summary of Research Methods for Medical Question and Answer Systems

Systematic synthesis of retrieved literature revealed prevailing research methodologies for medical question-answering (QA) systems, as cataloged in Table 3. Thematic trend analysis in Figure 6 further confirms deep learning and large language model (LLM) approaches as dominant methodologies through pronounced keyword bursts.

## 8. Conclusions

This paper systematically reviewed the research progress of medical QA systems. Existing literature provided the foundation for introducing fundamental concepts, definitions, and frameworks—progressing from traditional QA systems to medical QA system construction, with research advances across system modules examined. Secondly, combined with the actual needs of artificial intelligence in healthcare, it outlined the applications of medical QA systems in intelligent assisted diagnosis and treatment decision support, medical knowledge QA, traditional Chinese medicine research and development and pharmacy services, medical image report analysis for accurate diagnosis and treatment decisions, and quality control analysis of medical record content. Representative systems were detailed. In addition, a bibliometric analysis and discussion were presented, revealing the increasing research on the application of QA systems in healthcare.

Advancements in AI—particularly deep learning and NLP—are transforming medical QA systems from text-based to multimodal interaction, enabling more comprehensive and accurate medical information delivery. However, medical QA systems still have some problems and challenges in practical applications, which need to be further optimized and improved by researchers.

Technical evolution has established deep learning and large language models (LLMs) as dominant methodologies in medical QA systems. Domain-specific systems (e.g., WiNGPT with a 250 ms response time) outperform general models in targeted applications but require rigorous clinical validation for safety assurance. Multimodal diagnostics, TCM-oriented intelligent agents, and virtual reality surgery represent three high-potential research domains. However, their advancement hinges on resolving LLM compliance frameworks and adoption barriers in primary care settings.

Current research prioritizes multimodal diagnostic imaging, TCM agents, and VR-assisted surgery. Medical QA systems demonstrate continuous progress toward intelligence, personalization, and efficiency. Technological innovations will amplify their role in enhancing care quality, optimizing resource allocation, and accelerating medical AI integration.

This paper summarized these challenges and analyzed the future development direction of medical QA systems. Multimodal image diagnosis, Chinese medicine intelligence body, and virtual reality surgery are more likely to be favored by researchers in the future.

Medical QA system research is progressively advancing toward intelligent, personalized, and efficient solutions. Through ongoing innovation, these systems will serve increasingly critical roles in enhancing healthcare quality/efficiency, optimizing personalized resource allocation, and advancing medical AI applications.

Due to various factors, in the future, we will further study the research and application trends of question-answering systems in the medical field and further enrich the visual content of the article and strengthen the visual expression of future trend analysis.

## Figures and Tables

**Table 1:** Common biomedical datasets.

Name	Main Contents and Applications
Informatics for Integrating Biology and the Bedside (i2b2) [24]	Contains clinical text data, such as medical records, annotated with rich medical concepts and relationships, such as disease diagnosis and treatment. Used for clinical natural language processing tasks, such as information extraction, disease prediction, and optimization of electronic medical record systems.
Cochrane [25]	An important resource in the field of evidence-based medicine, systematically synthesizing the effectiveness and safety of various medical interventions. It provides a scientific basis for medical decision-making, clinical research, and health policy development, and helps researchers synthesize and analyze medical evidence.
MultiMedQA [26]	Multisource medical QA dataset that integrates several different medical QA datasets covering a wide range of medical topics and question types. Used to train more general and more generalized medical QA models for medical question-answering in different scenarios.
MedMCQA [27]	Contains multiple-choice questions and related knowledge points from medical exams, covering a wide range of aspects, including basic medicine and clinical practice. It is often used to train and evaluate medical knowledge quiz models and help in medical education and intelligent tutoring system development.
MIMIC-Ⅲ [28]	It is a clinical database for intensive care units (ICUs), containing detailed data on patients’ vital signs, laboratory tests, and medical orders. It is mainly used for medical research, such as disease prediction, evaluation of treatment effects, and development of clinical decision support systems.
BioASQ [29]	It is a question and answer dataset about the biomedical field, containing a large number of questions extracted from the literature and the corresponding answers. It is mainly used to train and evaluate biomedical QA systems to improve their accuracy and performance in answering complex medical questions.
Chest X-ray 14 [30]	Contains a large number of chest X-ray images and corresponding disease labels, labeled with 14 common chest diseases. Mainly used in the field of medical image analysis to train and evaluate deep learning models for chest disease diagnosis.
PubMed [31]	It has a huge amount of biomedical literature abstracts and part of the full text, covering the research results of medicine, biology, and other fields. Commonly used in medical knowledge retrieval, text mining, information extraction, and construction of medical knowledge bases and other research.
MedicationQA [32]	Contains QA data on drug information, medication recommendations, drug interactions, etc., centered around drug-related questions. It is used to train drug-related QA systems to assist clinicians and patients in making rational medication decisions.

**Table 2:** Comparison of mainstream healthcare QA systems.

System Name	Core Technology	Application Scenario	Accuracy	Typical Effectiveness
Shenglang AI	Deep Learning + NLP + Knowledge Graph	Clinical aids to diagnosis, patient counseling	92%	37% reduction in misdiagnosis rate
Zhiyun Health	Knowledge Graph + Rule Engine + Machine Learning	Retail pharmacy, hospital pre-screening	99.97%	Increased accuracy of medication risk warnings
MedAI	NLU + Medical Coding System + RWD Analytics	Drug development, clinical trial management	98.7%	15–20% reduction in development cycle time
WiNGPT	Large Models + Integrative Medicine Modelling	Full-cycle diagnosis and treatment, intelligent Chinese medicine	95%	Clinic time reduced by 22 min

**Table 3:** Summary of research methodologies for medical QA systems.

Category of Methods	Typical Representative Technologies
Rule-based methods	Information retrieval, pattern matching
Statistical machine-learning-based methods	Machine translation models (e.g., SMT, NMT, etc.), topic models (e.g., LDA)
Deep-learning-based methods	Pre-trained language models (e.g., BERT, GPT, etc.), knowledge graph embedding models (e.g., TransE, Node2Vec, etc.)
Large language modeling (LLM)	ChatGPT, MedGPT, LLaMA

## Data Availability

The original contributions presented in this study are included in the article. Further inquiries can be directed to the corresponding author.
